# sEMG Spectral Analysis and Machine Learning Algorithms Are Able to Discriminate Biomechanical Risk Classes Associated with Manual Material Liftings

**DOI:** 10.3390/bioengineering10091103

**Published:** 2023-09-20

**Authors:** Leandro Donisi, Deborah Jacob, Lorena Guerrini, Giuseppe Prisco, Fabrizio Esposito, Mario Cesarelli, Francesco Amato, Paolo Gargiulo

**Affiliations:** 1 Department of Advanced Medical and Surgical Sciences, University of Campania Luigi Vanvitelli, 80138 Naples, Italy; fabrizio.esposito@unicampania.it; 2 The Institute of Biomedical and Neural Engineering, School of Science and Engineering, Reykjavik University, 102 Reykjavik, Iceland; deborah20@ru.is (D.J.); lorena22@ru.is (L.G.); paolo@ru.is (P.G.); 3 Department of Engineering, University of Campania Luigi Vanvitelli, 81031 Aversa, Italy; 4 Department of Medicine and Health Sciences, University of Molise, 86100 Campobasso, Italy; g.prisco2@studenti.unimol.it; 5 Department of Engineering, University of Sannio, 82100 Benevento, Italy; mcesarelli@unisannio.it; 6 Department of Information Technology and Electrical Engineering, University of Naples Federico II, 80125 Naples, Italy; framato@unina.it; 7 Department of Science, Landspitali University Hospital, 102 Reykjavik, Iceland

**Keywords:** biomechanical risk assessment, load lifting, machine learning, physical ergonomics, Revised NIOSH Lifting Equation, surface electromyography, wearable devices, work-related musculoskeletal disorders

## Abstract

Manual material handling and load lifting are activities that can cause work-related musculoskeletal disorders. For this reason, the National Institute for Occupational Safety and Health proposed an equation depending on the following parameters: intensity, duration, frequency, and geometric characteristics associated with the load lifting. In this paper, we explore the feasibility of several Machine Learning (ML) algorithms, fed with frequency-domain features extracted from electromyographic (EMG) signals of back muscles, to discriminate biomechanical risk classes defined by the Revised NIOSH Lifting Equation. The EMG signals of the multifidus and erector spinae muscles were acquired by means of a wearable device for surface EMG and then segmented to extract several frequency-domain features relating to the Total Power Spectrum of the EMG signal. These features were fed to several ML algorithms to assess their prediction power. The ML algorithms produced interesting results in the classification task, with the Support Vector Machine algorithm outperforming the others with accuracy and Area under the Receiver Operating Characteristic Curve values of up to 0.985. Moreover, a correlation between muscular fatigue and risky lifting activities was found. These results showed the feasibility of the proposed methodology—based on wearable sensors and artificial intelligence—to predict the biomechanical risk associated with load lifting. A future investigation on an enriched study population and additional lifting scenarios could confirm the potential of the proposed methodology and its applicability in the field of occupational ergonomics.

## 1. Introduction

The risk of developing work-related musculoskeletal disorders (WRMDs) is strongly correlated with physical work, as shown in several studies [1,2,3,4]. Intensity, repetition, and duration are the three elements that have the greatest impact on biomechanical risk during manual tasks [5]. Biomechanical overload and the development of WRMDs mainly affect workers involved in activities such as manual material handling and load-lifting activities. Several quantitative and semi-quantitative observational methods have been proposed in occupational ergonomics [6,7,8,9,10,11,12] to evaluate the biomechanical risk exposure eventually coupled with dedicated software such as DELMIA [13]. In this context, the National Institute for Occupational Safety and Health (NIOSH) developed a methodology for evaluating biomechanical risk using a mathematical formula whose terms are function of intensity, duration, and frequency of the lifting activity as well as the geometric characteristics of the lifting task [11]. However, the various applications of these tools in actual ergonomic studies tend to be very time consuming and trivial due to the lack of a valid framework to guide the process [14]. For this reason, recently, as they emerge from the scientific literature, wearable sensors are spreading in the clinical setting and, in particular, in the context of occupational medicine and physical ergonomics [15,16,17,18,19], especially oriented to monitor work activities and to prevent WRMDs [20,21,22,23,24,25]. Among these instrumental methods, wearable sensors, which allow the acquisition of inertial signals such as linear acceleration and angular velocity; surface electromyographic (sEMG) signals; and pressure signals have proven to be useful to monitor workers’ activities.

Additionally, the use of these wearable systems coupled with artificial intelligence (AI) is also growing in the ergonomic field, and several studies have been proposed in the scientific literature. For instance, Conforti et al. [26] proposed a methodology to recognize safe and unsafe postures through wearable sensors and Machine Learning (ML) algorithms fed with kinematic features extracted from linear acceleration and angular velocity signals. They used a Support Vector Machine (SVM) algorithm reaching an accuracy to classify safe and unsafe posture equal to 99.4%. Estrada et al. [27] studied the feasibility of a Decision Tree (DT) algorithm, fed with features extracted from a bending signal acquired by flex sensors, to recognize proper and improper sitting postures with a laptop, reaching an accuracy equal to 80%. Olsen et al. [28] analysed the discrimination power of ML and Deep Learning (DL) algorithms to classify correct and incorrect postures of dental practitioners using features extracted from inclinometer data. The best algorithm was k-Nearest Neighbour (kNN) that reached an accuracy equal to 99.94%. Antwi-Afari et al. [29] implemented several ML algorithms fed with features extracted from foot plantar pressure and linear acceleration with the aim to detect and classify awkward working postures. The best classifier was SVM that showed an accuracy equal to 99.90%. Zhang et al. [30] used an SVM to recognize jerk changes due to physical exertion using jerk-based features extracted from 17 Inertial Measurement Units (IMUs) placed on several points on the subject’s body. Donisi et al. [31] proposed a new methodology to discriminate biomechanical risk classes—defined according to the Revised NIOSH Lifting Equation (RNLE)—using a wearable inertial sensor and ML algorithms. They used only one IMU placed on the lumbar region and extracted several time-domain features from the acquired inertial signals; Random Forest (RF) was the best classification algorithm with an accuracy and an Area Under the Receiving Characteristic Curve (AUCROC) greater than 90% and 94%, respectively. Aiello et al. [32] studied the kNN feasibility—fed with time-domain features extracted from linear acceleration acquired by two accelerometers placed on the wrists—to classify heavy-duty and hard-duty activities associated with exposure to vibrations; the kNN algorithm reached an accuracy equal to 94%. Zhao et al. [33] proposed a DL algorithm, namely, Convolutional Long Short-Term Memory (CLSTM), able to recognize workers’ posture according to the Ovako Working posture Assessment System (OWAS) criteria using inertial data acquired by means of IMUs placed on the forehead, chest, arm, thigh, and calf. Umer et al. [34] studied the feasibility of several ML algorithms to predict the physical exertion level using multiple physiological measures (ECG, skin temperature, respiration) according to the Borg-20 scale. Yu et al. [35] calculated the workload and planned ergonomic risks’ mitigation strategies using computer vision, IMU sensors, and pressure insoles. Mudiyanselage et al. [36] proposed a methodology to detect the level of risk of harmful lifting activities, defined by NIOSH, using ML and DL algorithms fed with features extracted from thoracic and multifidus sEMG signals, while Donisi et al. [37] studied the classification power of some ML algorithms to discriminate biomechanical risk classes, defined according to the RNLE, using features extracted from the bicep muscle.

Considering the increasing use of wearable sensors and AI in the physical ergonomic field, the aim of the present work was to study the feasibility of several ML algorithms—fed with specific frequency-domain features extracted from the Total Power Spectrum (TPS) of the sEMG signals of the erector spinae and multifidus muscles—to classify biomechanical risk classes defined by the RNLE. Moreover, the correlation between muscular fatigue and risky weightlifting activities was explored. The proposed methodology, which, to the best of the authors’ knowledge, is novel in the literature, provides a new tool—based on ML algorithms—to assess the biomechanical risk associated with load lifting. This methodology could overcome the limits of observational methods, nowadays considered the gold standard, that are often time-consuming and/or depend on the operator’s experience and skill.

## 2. Materials and Methods

### 2.1. Wearable System for Surface Electomiography—The KineLive System

**Figure 1:**
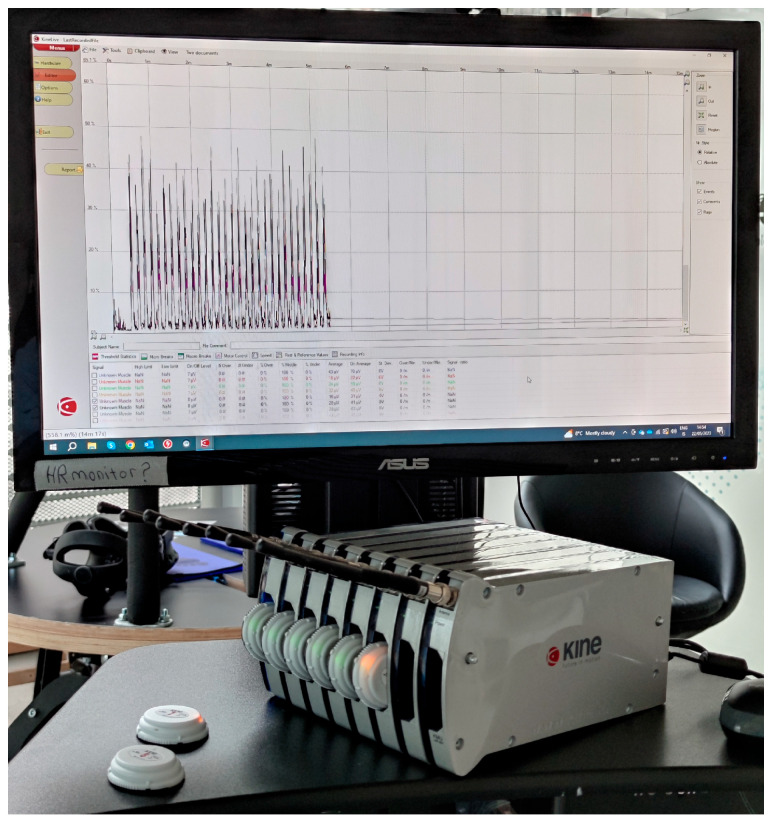
sEMG sensors and their case; on the monitor, the EMG signal is managed by the dedicated software.

**Figure 2:**
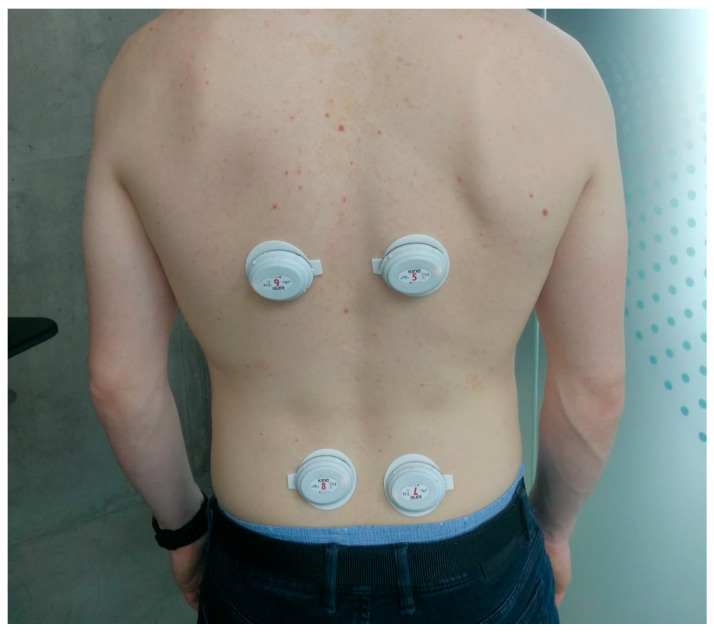
Placement of the four sEMG sensors: 2 sensors placed on the right and left erector spinae muscles and 2 sensors placed on the right and left multifidus muscles.

The KineLive System (Kiso Ehf Inc., Reykjavík, Iceland) is a commercial wearable system for sEMG widely used in the scientific literature [38,39]. The wireless EMG sensors are capable of capturing muscle electrical activities at a sampling frequency of 1600 Hz. The sensors store the data locally during acquisition. Following the end of the experiment, the stored data are uploaded by radiofrequency to the KineLive software (shown in Figure 1). Upon replacement into the charging station, any data on the sensors are deleted. Figure 2 illustrates the placement of four sensors, with two positioned on the upper back and two on the lower part of the participant. These sensors are specifically targeting the erector spinae and multifidus muscles.

### 2.2. Revised NIOSH Lifting Equation

The RNLE is a methodology widely used in occupational ergonomics to assess the potential biomechanical risk associated with manual material handling and lifting activities [40,41]. The equation allows us to calculate the Recommended Weight Limit (RWL) as a function of specific characteristics of lifting as reported in (1):

RWL = LC × HM × VM × DM × AM × FM × GM,(1)

where:

- LC: Load Constant 25/20 kg (males, <45/>45 years old, respectively), 20/15 kg (females, <45/>45 years old, respectively);
- HM: Horizontal Multiplier;
- VM: Vertical Multiplier;
- DM: Distance Multiplier;
- AM: Asymmetric Multiplier;
- FM: Frequency Multiplier;
- GM: Grab Multiplier.

After computing the RWL, the Lifting Index (LI) is computed, dividing the weight lifted by the RWL. The LI is a measure of the potential biomechanical risk associated with the weightlifting [42]. A lifting associated with a LI < 1 is defined as acceptable or safe, while a LI > 1 is indicative of a risky lifting with a potential biomechanical risk that increases as the value of the LI increases.

### 2.3. Study Population

The study was carried out on a study population composed of 8 subjects (7 male, 1 female) whose anthropometric characteristics are reported in Table 1. The study was conducted at the Motion Sickness Laboratory of the Reykjavik University. The selected participants were not affected by musculoskeletal disorders or other occupational pathologies. The study was approved by the local Ethics Committee in accordance with the Declaration of Helsinki, and all the participants signed a declaration of informed consent.

### 2.4. Study Protocol

Each participant carried out a task session based on two different trials. The first trial consisted of 30 consecutive load-lifting tasks in a condition with a LI < 1 (LI = 0.5) defined as a NO-RISK class, while the second trial consisted of 30 consecutive load-lifting tasks in a condition with a LI > 1 (LI = 1.3) defined as a RISK class. Table 2 shows the parameter values used to compute the LI.

**Figure 3:**
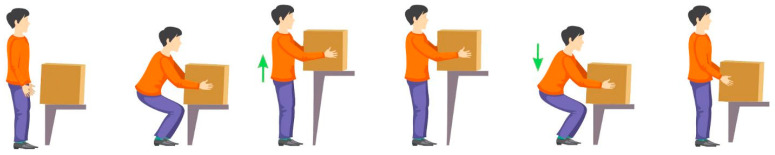
Phases of a load-lifting task.

The trial was performed using a plastic container with ergonomic handles and weights equally distributed inside. The participants performed the lifting task adopting the squat technique (safe posture) with a two-handed grip as shown in Figure 3.

### 2.5. Digital Signal Processing and Feature Extraction

**Figure 4:**
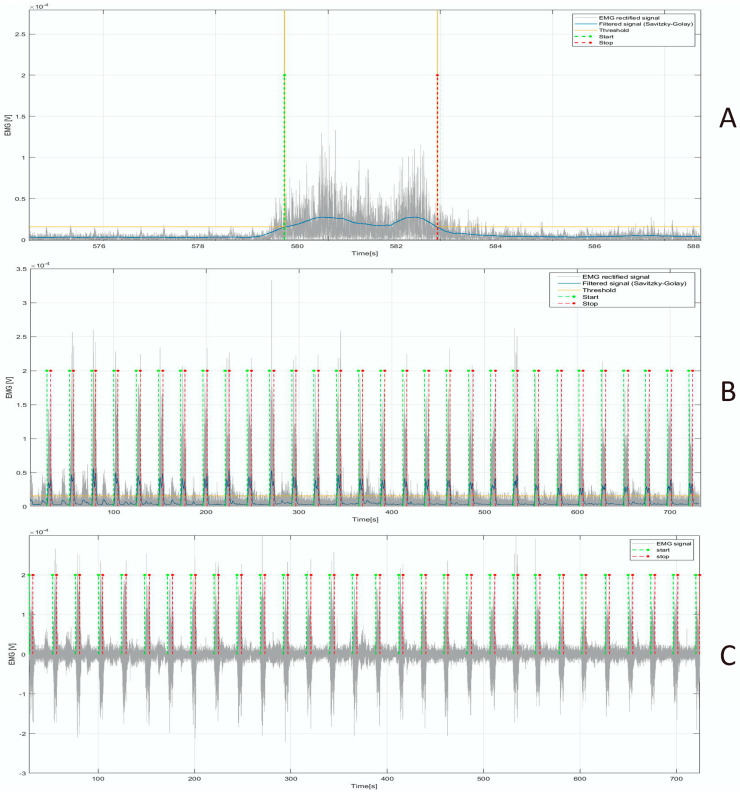
(**A**) Rectified original signal (in grey), rectified and filtered signal by means of Savitzky–Golay filter (in blue), and threshold (in yellow) to determine the start and stop points (in green and red, respectively). A single lifting is illustrated. (**B**) Rectified original signal (in grey), rectified and filtered signal by means of Savitzky-Golay filter (in blue), and threshold (in yellow) to determine the start and stop points (in green and red, respectively). All the liftings of a single trial are illustrated. (**C**) Original EMG signal and start and stop points employed to identify the ROIs.

The EMG signals acquired from the erector spinae and multifidus muscles underwent a digital signal processing consisting of filtering and segmentation in order to extract the Regions of Interest (ROIs) within which to carry out a feature extraction in the frequency-domain. EMG signals were filtered with an 8th-order Butterworth filter, with a band pass ranging from 15 to 400 Hz. Successively, the signals were rectified and then filtered with a 4th-order Butterworth low-pass filter with a cut-off frequency equal to 20 Hz. Finally, the resulting signals were filtered by means of a Savitzky–Golay filter [43] with a third-order polynomial and a frame length equal to 3001. An empirical threshold was applied to compute the starting and ending points of the window time during which the participant performed the lifting task (Figure 4A,B), and therefore, the ROIs corresponding to the muscle contraction were selected from the original EMG signal (Figure 4C).

For each ROI of the EMG signal, the TPS was computed using the fast Fourier transform (fft) algorithm. From the TPS, the following features were extracted:

- Total power (Power) [V^2^]: the integral under the spectrum curve;
- Peak power (P_power) [V^2^/Hz]: the maximum value of the TPS;
- Median frequency (F_median) [Hz]: the frequency that divides the total power area into two equal parts;
- Mean frequency (F_mean) [Hz]: the mathematical mean of the spectrum curve;
- Peak frequency (F_peak) [Hz]: the frequency at which the P_power is attained;
- Kurtosis (adimensional): the standardized fourth moment of a distribution that represents a measure of the tailedness of a given distribution;
- Skewness (adimensional): the third standardized moment of a distribution that represents a measure of the asymmetry of a given distribution.

### 2.6. Statistical Analysis

A Shapiro–Wilk normality test was performed to assess the normality of each feature before to carry out a parametric (*t*-test) or non-parametric (Wilcoxon test) two-tailed paired test. This statistic test was used to verify whether each feature was differentiated in a statistically significant way between the condition with LI < 1 (NO-RISK class) and the condition with LI > 1 (RISK class). For all the statistical tests, a confidence level equal to 95% was chosen (definition of statistical significance: *p*-value < 0.05).

### 2.7. Machine Learning Analysis

ML is a field of inquiry devoted to understanding and building models that leverage data to improve performance regarding a set of tasks. It uses statistical techniques and advanced algorithms to improve the accuracy of prediction.

Several ML algorithms were implemented to assess the feasibility of the proposed methodology to discriminate biomechanical risk classes according to the RNLE. The ML classification algorithms analysed are illustrated below.

DT is a classifier algorithm that represents the simplest and the most used logic-based classification technique. The algorithm classifies data by ordering them as trees on the basis of their characteristic values [44]. The following hyperparameters of DT J48 were set: confidence factor equal to 0.25, minimum number of instances per leaf equal to 2, and seed equal to 1; the pruning was not implemented.

Gradient Boost Tree (GBT) is a boosting algorithm whose purpose is to reduce the loss of the function model by adding learning weaknesses and using gradient descent to establish the local minimum of the differential function [45]. For this algorithm, the limit number of levels (tree depth) was set equal to 5, and the number of models and the learning rate were set to 92 and 0.832, respectively.

The kNN algorithm ranks the unlabelled instance vector according to the label class that has the majority among its closest k neighbours in the training set [46]. A k equal to 11 was set, and a Euclidean distance was computed.

Naive Bayes (NB) is a probabilistic ML method. The probability of each class for a given instance is calculated, and the class with the highest probability is then returned [47]. Default probability, minimum standard deviation, threshold standard deviation, and maximum number of unique nominal values per attribute were set to 0.0001, 0.068, 0.006, and 33, respectively.

An SVM in a binary classification—as in the case under study—finds a hyperplane that divides data from two different classes. The largest possible distance is established between the separating hyperplane by maximizing the margin, thus creating the separation [48]. A polynomial kernel with power equal to 1.882, bias equal to 1.894, and gamma equal to 1.495 was chosen.

Logistic Regression (LR) iteratively identifies the strongest linear combination of features with the highest probability to detect the observed outcome [49]. As a solver algorithm, the stochastic average gradient was selected. The maximum number of epochs and the parameter epsilon were set to 197 and 0.005, respectively. The learning rate strategy was considered fixed, and the step size was set to 1.886.

For all the ML algorithms, the hyperparameters optimization was carried out in order to maximize the classification accuracy. Moreover, for the LR, kNN, and SVM algorithms, the min–max normalization was performed.

As a validation strategy, the ten-fold cross-validation (CV) was adopted. It consists of splitting the dataset into ten subsets, with the iterative use of nine of them to train the model and the final one to evaluate its performance [50]. Moreover, a stratified CV was performed in order to keep the proportions between the two classes unaltered among the folds [51].

Moreover, another validation strategy was adopted to better generalize the validation of our proposed approach, namely, the leave-one-subject-out CV. Seven subjects were used to train the predictive models and one subject to test the algorithms, and this was conducted iteratively eight times.

The feasibility of the proposed ML algorithms was evaluated using the following metrics: accuracy, F-measure, specificity, sensitivity, precision, recall, and AUCROC [52]. Moreover, the confusion matrix of the best algorithms was also reported.

A feature importance by means of the calculation of the Information Gain (IG) was computed. The IG is an indicator of the amount of information provided by the features [53].

Finally, a trend analysis using a linear regression model was carried out in order to assess whether any trends of the parameters, namely, F_mean and F_median, occurred, since several works in the scientific literature have shown a correlation between muscular fatigue and a change in the spectrum of the EMG signal [54,55,56,57].

The ML analysis was performed using the Knime Analytics Platform, a platform widely used in the biomedical engineering field [58,59,60,61,62,63].

## 3. Results

Firstly, a statistical analysis by means of a parametric (*t*-test) or non-parametric (Wilcoxon test) two-tailed paired test was carried out in order to assess if each feature was differentiated in a statistically significant way between the two risk classes, namely, NO-RISK and RISK. This analysis was performed separately for the erector spinae muscle and the multifidus muscle. Table 3 and Table 4 show the results of the two-tailed paired test for the erector spinae and multifidus muscle, respectively.

Secondly, an ML analysis was carried out to assess the feasibility of the ML algorithms fed with frequency-domain features extracted from the TPS of the EMG to discriminate biomechanical risk classes defined by means of the RNLE. Table 5 shows the evaluation metric scores reached by the ML algorithms using the ten-fold cross-validation and considering both muscles. In Table 6, the confusion matrix of the best algorithms, namely, SVM, is reported.

Thirdly, to obtain more robust and generable results, we performed a ML analysis using the leave-one-subject-out CV using N-1 subjects to train the classifiers and one subject to test and repeat this procedure N times (with N denoting the number of subjects that compose the study population). The results of this further ML analysis are reported in Table 7.

**Figure 5:**
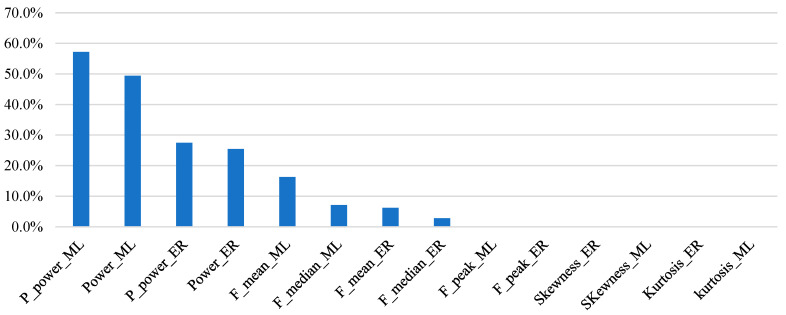
Ranking of the features extracted from EMG signals acquired from multifidus and erector spinae muscles according to the IG approach.

Moreover, a feature importance by means of the calculation of the IG was performed, and the results are showed in Figure 5.

**Figure 6:**
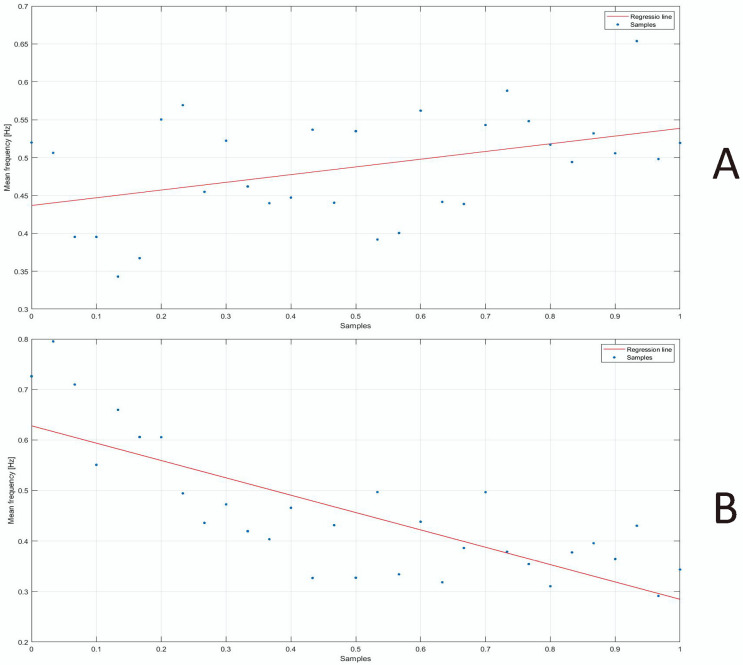

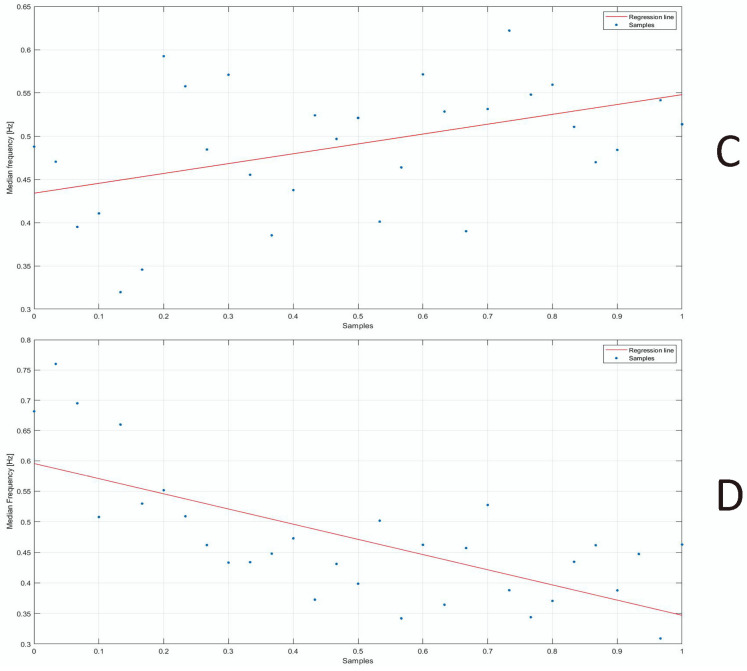
(**A**) Mean frequency trend relating to multifidus muscle (NO-RISK) (m = 0.122, q = 0.460). (**B**) Mean frequency trend relating to multifidus muscle (RISK) (m = −0.343, q = 0.628). (**C**) Median frequency trend relating to multifidus muscle (NO-RISK) (m = 0.102, q = 0.437). (**D**) Median frequency trend relating to multifidus muscle (RISK) (m = −0.215, q = 0.574).

Finally, a trend analysis was carried out in order to study the effect of the muscular fatigue occurring during the liftings in the two study conditions, namely, NO-RISK and RISK. Figure 6 reports the trend analysis for the multifidus F_mean and multifidus F_median in both conditions, namely, RISK and NO-RISK. For the sake of brevity, the same plots for the erector spinae are not reported, but they are discussed in the Discussion section.

## 4. Discussion

The objective of this work was to explore the feasibility of several ML algorithms fed with frequency-domain features extracted from the TPS of the EMG of back muscles (multifidus and erector spinae) to discriminate biomechanical risk classes associated with load lifting according to the RNLE.

The results reported in Table 3 and Table 4 show that all the frequency-domain features extracted from the TPS of the multifidus muscle exhibited a statistically significant difference between the two classes (almost always, the maximum statistically significant difference *p*-value is lower than 0.001), namely, NO RISK and RISK, while for the erector spinae muscle, only the F_peak feature did not exhibit a statistically significant difference with a *p*-value equal to 0.523. This former statistical analysis allows us to suppose that the multifidus muscle is more predictive of the biomechanical risk classes compared to the erector spinae. This result may be associated with the fact that lower back muscles are more stressed in load-lifting tasks compared to the dorsal ones; in fact, most of the musculoskeletal disorders associated with lifting loads affect the lumbar region.

Table 5 reports the scores reached from each ML algorithm, and the feasibility of the ML (except NB) algorithms coupled with the extracted features to discriminate the two biomechanical risk classes is highlighted. The low performances of the NB could be due to the correlation existing between instances and between features, since the NB algorithm needs to be fed with non-correlated features. The best ML algorithm was SVM with an accuracy, F-measure, specificity, sensitivity, precision, recall, and AUCROC equal to 0.961, 0.962, 0.955, 0.967, 0.956, 0.967, and 0.985, respectively. Conventionally, AUCROC values greater than 0.70 are indicative of a moderate discrimination power of the algorithm, values greater than 0.80 show a good discrimination, while values greater than 0.90 underline an excellent discrimination. The SVM fed with the extracted features reached an AUCROC equal to 0.961, demonstrating its excellent discrimination power for this specific task. Moreover, as shown in the confusion matrix (Table 6), the SVM classifier misclassified only 19 out of 492 instances.

Considering the correlation existing among the instances (liftings) of the same subject, a further ML analysis was performed in order to obtain more robust results. Table 7 shows the metrics’ score of the ML algorithms using the leave-one-subject-out CV; in this way, no instances related to a single subject are present in both the training and the test set. SVM was the best ML algorithm once again with an accuracy and AUCROC equal to 0.848 and 0.918 (in mean value), respectively.

The ML analyses proved that the SVM algorithm coupled with the extracted frequency-domain features is a valid tool to discriminate biomechanical risk classes associated with load lifting, since the AUCROC values are always greater than 90%.

The feature-importance analysis shown in Figure 5 highlighted the discriminative power of the following features: P_power, power, F_mean, and F_median of both muscles. P_power and power are strictly correlated with the force of contraction, while F_mean and F_median are correlated with muscular fatigue [64]. These results are in line with the interpretation that increasing the risk (performing a lifting task with an LI greater than one) increases the force of the contraction and the muscular fatigue. In fact, looking at Table 3 and Table 4, the power and P_power increase with higher LIs, while the F_mean and F_median decrease with higher LIs because muscular fatigue takes over. Therefore, these features were demonstrated to have a predictive value to discriminate biomechanical risk classes. Moreover, as shown in Figure 5, the features related to the multifidus muscle are more important than the features related to the erector spinae muscle (67.5% and 32.5%, respectively); these results are in line with the results of the statistical analysis, underlining the impact of risky load-lifting activities on injuries associated with the lumbar region.

Different studies presented in the scientific literature have attempted to classify biomechanical risk classes according to the RNLE using ML algorithms. Donisi et al. [31] proposed a methodology to discriminate biomechanical risk classes according to the RNLE using time-domain features extracted from inertial signals (acceleration and angular velocity) acquired by means of an IMU placed on the lumbar zone, reaching accuracy and AUCROC values greater than 90% and 94%, respectively. In another study, Donisi et al. [65] explored the feasibility of a logistic regression model fed with time- and frequency- domain features extracted from signals acquired using one IMU placed on the sternum to classify risk classes associated with lifting activities according to the RNLE, reaching an accuracy equal to 82.8%. Differently from the present study, the authors used inertial signals.

Similarly, an approach using sEMG signals was proposed by Mudiyanselage et al. [36] that studied the feasibility of several ML algorithms fed with time-domain features extracted from sEMG signals of the multifidus and the thoracic muscles. Contrary to our work, in [36], the best ML algorithm was the DT that reached an accuracy equal to 99.96%. In the study of Donisi et al. [37], the authors studied the feasibility of time-domain and frequency-domain features extracted from bicep EMG signals to discriminate risk/no-risk conditions according to the RNLE, reaching evaluation metrics’ scores greater than 95%, but they investigate only tree-based ML algorithms, and they did not investigate the muscular fatigue. Moreover, they considered a very small study population, and therefore, the results cannot be considered robust. Finally, they focused on the bicep muscle, which is not subject to injury as much as back muscles during load-lifting activities.

In the study by Varecchia et al. [66], the combined use of an artificial neural network fed with time-domain and frequency-domain features, extracted from sEMG and optoelectronic systems, resulted in a classification accuracy of up to 90% in discriminating three NIOSH risk classes (LI = 1, LI = 2, LI = 3). In another work by the same authors [67], a new feature named Lifting Energy Consumption [68] was used to feed a neural network, demonstrating an accuracy of up to 100%. The limitation of the methodology proposed by the authors, as highlighted by the authors themselves, is due to the poor applicability in the workplace due to the high number of sensors (both sEMG sensors and markers of an optoelectronic system). This configuration confines the biomechanical risk assessment to the laboratory. However, this issue can be solved by using the methodology proposed in this paper, where just two wearable sensors for sEMG are adopted.

The analysis of the EMG during fatiguing effort is a field of interest to many researchers. In occupational ergonomics, researchers and practitioners are concerned with workplace fatigue and the extent to which normal everyday activities may result in muscular fatigue that can be documented using EMG. Neck pain, shoulder pain, and back pain are serious problems for many professions, and there are many applications in ergonomics regarding the use of EMG to explore neck and low-back pain. Indeed, the EMG signal and the related parameters change with muscular fatigue in several muscles [64].

Great attention has been given to understand the changes in frequency characteristics of the EMG signal during fatiguing effort. Among these characteristics, the principal ones investigated in the scientific literature have been the F_mean and the F_median extracted from the TPS of the EMG signal [69].

Changes in the F_mean and F_median are both valuable, though they are highly intercorrelated. The decline in F_mean and F_median with fatigue is a common observation, since it has been seen in a wide variety of muscles [70,71,72] and has been verified by many researchers in numerous static and dynamic conditions.

On the basis of the results highlighted in the above-mentioned articles about the link between muscular fatigue and the change in the TPS of the EMG, the trend analysis presented in Figure 6 shows a negative trend in the F_mean and F_median for the lifting tasks performed in the RISK condition, while the trends in the same parameters for the lifting tasks performed in the NO-RISK condition showed a non-significant positive trend. This result suggests that during RISK liftings, muscle fatigue occurs, underling the predictive value of these two features to discriminate RISK/NO-RISK lifting activities.

Based on these results, the proposed methodology—which combine ML algorithms and features extracted from the TPS of EMG back muscles—proved to be able to discriminate biomechanical risk classes associated to lifting activities according to the RNLE.

## 5. Conclusions

The proposed methodology, based on AI and wearable sensors for sEMG, showed its feasibility—although preliminary in view of the small study population and lifting scenarios—to discriminate biomechanical risk classes associated with manual handling and, in particular, with load lifting. The study results show that the SVM algorithm was the best one able to classify biomechanical risk classes—defined by means of the RNLE—with accuracy and AUCROC values up to 0.985. This procedure allows biomechanical risk assessment in an automatic, economic, non-time-consuming, non-operator-dependent, and non-invasive way and therefore could be of direct practical relevance for occupational ergonomics. Moreover, the limited number and type of sensors renders the procedure of biomechanical risk assessment also suitable in the workplace and not confined to the laboratory, such as the other methodologies proposed in the scientific literature. Although combining different types of sensors can give more information about the worker’s risk level [73], the use of a single type of sensor, as in the case under study, can make the procedure more usable in the workplace. This paper has some limitations: limited sample size, age range too narrow, and imbalanced gender. Indeed, in this paper, we focused on a small, young study population (eight subjects) without any pathologies, and we did not consider individuals of advanced age with comorbidities and/or bone fragility, conditions that can trigger or aggravate WRMDs; indeed, older workers, as a function of having spent longer time in the job, are more susceptible to these conditions as a result of cumulative exposure. Moreover, we considered an unbalanced study population (seven males and one female), and it is demonstrated that women exhibit a higher incidence and prevalence of WRMDs than men in association with anthropometric differences. Finally, other sociodemographic variables, such as ethnicity, sex, age, and economic status, can be directly related to the onset of WRMDs, and therefore, they should be taken into account. Future investigations on a larger (in terms of age and samples) and gender-balanced study population and more lifting scenarios could confirm the potentiality of the proposed methodology and its feasibility to be integrated with methodologies used in the physical ergonomics field in order to assess the biomechanical risk to which workers are subject during manual material handling.

## Figures and Tables

**Table 1:** Anthropomorphic characteristics of the study population reported as mean ± standard deviation.

Characteristics	
Age (years)	24.50 ± 3.25
Height (cm)	181.75 ± 4.23
Weight (kg)	80.38 ± 8.52
Body Mass Index (kg/m^2^)	24.40 ± 3.28

**Table 2:** Combination of weight, frequency, and vertical displacement variables for lifting activities corresponding to LI < 1 and LI > 1 according to the RNLE.

Trial 1 (LI < 1, LI = 0.5)				Trial 2 (LI > 1, LI = 1.3)				
Vertical Displacement (cm)	Frequency (lifts/min)	Weight Lifted (kg)		Vertical Displacement (cm)	Frequency (lifts/min)		Weight Lifted (kg)	
M & F	M & F	M	F	M & F	M	F	M	F
50–120	2.5	7	5	50–120	6	4	15	10

M: male, F: female.

**Table 3:** Paired test between NO-RISK/RISK classes for each feature extracted from the EMG signal acquired from the erector spinae muscle.

Features	NO-RISK Mean ± Std	RISK Mean ± Std	*p*-Value
Power_ER	8.316 × 10^−9^ ± 7.216 × 10^−9^	1.084 × 10^−8^ ± 6.612 × 10^−9^	<0.001
P_power_ER	1.205 × 10^−10^ ± 7.835 × 10^−11^	1.891 × 10^−10^ ± 1.037 × 10^−10^	<0.001
F_peak_ER	48.611 ± 11.562	47.975 ± 11.226	0.523
F_median_ER	66.192 ± 10.160	64.776 ± 9.057	<0.001
F_mean_ER	79.215 ± 11.387	77.833 ± 10.237	<0.001
Kurtosis_ER	71.608 ± 37.604	74.134 ± 37.724	0.011
Skewness_ER	6.787 ± 1.361	6.954 ± 1.257	0.001

Definition of statistical significance: *p*-value < 0.05. ER: erector spinae.

**Table 4:** Paired test between NO-RISK/RISK classes for each feature extracted from the EMG signal from the multifidus muscle.

Features	NO-RISK Mean ± Std	RISK Mean ± Std	*p*-Value
Power_ML	1.095 × 10^−8^ ± 6.919 × 10^−9^	1.881 × 10^−8^ ± 1.326 × 10^−8^	<0.001
P_power_ML	1.298 × 10^−10^ ± 8.016 × 10^−11^	2.896 × 10^−10^ ± 2.435 × 10^−10^	<0.001
F_peak_ML	51.650 ± 16.828	48.935 ± 16.022	0.004
F_median_ML	78.600 ± 15.788	74.606 ± 17.650	<0.001
F_mean_ML	97.406 ± 15.216	92.443 ± 17.717	<0.001
Kurtosis_ML	66.686 ± 37.298	75.974 ± 48.0.69	<0.001
Skewness_ML	6.347 ± 1.518	6.781 ± 1.828	<0.001

Definition of statistical significance: *p*-value < 0.05. ML: multifidus.

**Table 5:** Evaluation metric scores using features extracted from EMG signals (both erector spinae and multifidus), k-fold cross-validation strategy (k = 10) and optimization parameters for each classification algorithm.

	SVM	KNN	DT	GR Boost	LR	NB
Accuracy	0.961	0.907	0.715	0.943	0.85	0.671
F-measure	0.962	0.91	0.735	0.942	0.857	0.719
Specificity	0.955	0.866	0.642	0.963	0.801	0.500
Sensitivity	0.967	0.947	0.789	0.923	0.898	0.841
Precision	0.956	0.876	0.688	0.962	0.819	0.627
Recall	0.967	0.947	0.789	0.923	0.898	0.841
AUCROC	0.985	0.961	0.712	0.987	0.910	0.782

**Table 6:** Confusion matrix of the best classification algorithm (SVM).

	NO-RISK	RISK
NO-RISK	238	8
RISK	11	235

**Table 7:** Evaluation metric scores using features extracted from EMG signals (both erector spinae and multifidus), leave-one-subject-out CV strategy and optimization parameters for each classification algorithm.

	SVM	KNN	DT	GR Boost	LR	NB
Accuracy	0.848 ± 0.068	0.787 ± 0.102	0.522 ± 0.153	0.647 ± 0.189	0.785 ± 0.066	0.553 ± 0.067
F-measure	0.855 ± 0.059	0.728 ± 0.187	0.509 ± 0.212	0.687 ± 0.209	0.776 ± 0.082	0.518 ± 0.193
Specificity	0.806 ± 0.148	0.923 ± 0.084	0.448 ± 0.405	0.483 ± 0.398	0.794 ± 0.165	0.488 ± 0.476
Sensitivity	0.890 ± 0.095	0.650 ± 0.240	0.583 ± 0.330	0.812 ± 0.288	0.776 ± 0.168	0.618 ± 0.408
Precision	0.836 ± 0.102	0.909 ± 0.078	0.585 ± 0.226	0.638 ± 0.215	0.817 ± 0.118	0.653 ± 0.206
Recall	0.890 ± 0.095	0.650 ± 0.240	0.583 ± 0.330	0.812 ± 0.288	0.776 ± 0.168	0.618 ± 0.408
AUCROC	0.918 ± 0.106	0.907 ± 0.074	0.559 ± 0.200	0.735 ± 0.207	0.839 ± 0.087	0.674 ± 0.127

## Data Availability

The datasets generated and analysed in this study are not publicly available due to privacy policy but are available from the corresponding author on reasonable request.

## References

[1] Radwin R.G., Marras W.S., Lavender S.A. (2001). Biomechanical aspects of work-related musculoskeletal disorders. Theor. Issues Ergon. Sci..

[2] Keyserling W.M., Brouwer M., Silverstein B.A. (1992). A checklist for evaluating ergonomic risk factors resulting from awkward postures of the legs, trunk and neck. Int. J. Ind. Ergon..

[3] Marras W.S., Lavender S.A., Leurgans S.E., Fathallah F.A., Ferguson S.A., Gary Allread W., Rajulu S.L. (1995). Biomechanical risk factors for occupationally related low back disorders. Ergonomics.

[4] Punnett L., Fine L.J., Keyserling W.M., Herrin G.D., Chaffin D.B. (1991). Back disorders and nonneutral trunk postures of automobile assembly workers. Scand. J. Work Environ. Health.

[5] Trask C., Mathiassen S.E., Wahlström J., Heiden M., Rezagholi M. (2012). Modeling costs of exposure assessment methods in industrial environments. Work.

[6] Buchholz B., Paquet V., Punnett L., Lee D., Moir S. (1996). PATH: A work sampling-based approach to ergonomic job analysis for construction and other non-repetitive work. Appl. Ergon..

[7] Karhu O., Kansi P., Kuorinka I. (1977). Correcting working postures in industry: A practical method for analysis. Appl. Ergon..

[8] Lynn M., Corlett N. (1993). RULA: A survey method for the investigation of work-related upper limb disorders. Appl. Ergon..

[9] Hignett S., McAtamney L. (2000). REBA: A survey method for the investigation of work-related upper limb disorders. Appl. Ergon..

[10] Battevi N., Menoni O., Ricci M.G., Cairoli S. (2006). MAPO index for risk assessment of patient manual handling in hospital wards: A validation study. Ergonomics.

[11] Walter T.R., Putz-Anderson V., Garg A., Lawrence J. (1993). Revised NIOSH equation for the design and evaluation of manual lifting task. Ergonomics.

[12] Takala E.P., Pehkonen I., Forsman M., Hansson G.Å., Mathiassen S.E., Neumann W.P., Sjøgaard G., Veiersted K.B., Westgaard R.H., Winkel J. (2010). Systematic evaluation of observational methods assessing biomechanical exposures at work. Scand. J. Work Environ. Health.

[13] Emodi C.T. (2007). A general Computer-Based Methodology for Work Injury Analysis in a Production Assembly Line. Ph.D. Dissertation.

[14] Emodi C.T., Zhang W.J., Lang S.Y., Bi Z.M. (2007). A Framework for Modeling and Analysis of Human Repetitive Operations in a Production/assembly Line (No. 2007-01-2500).

[15] Donisi L., Amitrano F., Coccia A., Mercogliano L., Cesarelli G., D’Addio G. (2021). Influence of the Backpack on School Children’s Gait: A Statistical and Machine Learning Approach. Proceedings of the 8th European Medical and Biological Engineering Conference (EMBEC 2020).

[16] Onofrejova D., Balazikova M., Glatz J., Kotianova Z., Vaskovicova K. (2022). Ergonomic assessment of physical load in Slovak industry using wearable technologies. Appl. Sci..

[17] Chander H., Burch R.F., Talegaonkar P., Saucier D., Luczak T., Ball J.E., Turner A., Kodithuwakku Arachchige S.N.K., Carroll W., Smith B.K. (2020). Wearable stretch sensors for human movement monitoring and fall detection in ergonomics. Int. J. Environ. Res. Public Health.

[18] Lind C.M., Diaz-Olivares J.A., Lindecrantz K., Eklund J. (2020). A wearable sensor system for physical ergonomics interventions using haptic feedback. Sensors.

[19] Tsao L., Li L., Ma L. (2018). Human work and status evaluation based on wearable sensors in human factors and ergonomics: A review. IEEE Trans. Hum.-Mach. Syst..

[20] Donisi L., Cesarelli G., Pisani N., Ponsiglione A.M., Ricciardi C., Capodaglio E. (2022). Wearable Sensors and Artificial Intelligence for Physical Ergonomics: A Systematic Review of Literature. Diagnostics.

[21] Lopez-Nava I.H., Munoz-Melendez A. (2016). Wearable inertial sensors for human motion analysis: A review. IEEE Sens. J..

[22] Hafez K. (2022). Input Variables for Manual Material Handling Assessment Methods Obtained Using Body Worn Sensors. J. Ergon..

[23] Chan V.C., Ross G.B., Clouthier A.L., Fischer S.L., Graham R.B. (2022). The role of machine learning in the primary prevention of work-related musculoskeletal disorders: A scoping review. Appl. Ergon..

[24] Ranavolo A., Draicchio F., Varrecchia T., Silvetti A., Iavicoli S. (2018). Wearable monitoring devices for biomechanical risk assessment at work: Current status and future challenges—A systematic review. Int. J. Environ. Res. Public Health.

[25] Stefana E., Marciano F., Rossi D., Cocca P., Tomasoni G. (2021). Wearable devices for ergonomics: A systematic literature review. Sensors.

[26] Conforti I., Mileti I., Del Prete Z., Palermo E. (2020). Measuring biomechanical risk in lifting load tasks through wearable system and machine-learning approach. Sensors.

[27] Estrada J.E., Vea L.A. Real-Time Human Sitting Position Recognition Using Wireless Sensors. Proceedings of the 2020 2nd International Conference on Image, Video and Signal Processing.

[28] Olsen G.F., Brilliant S.S., Primeaux D., Najarian K. Signal processing and machine learning for real-time classification of ergonomic posture with unobtrusive on-body sensors; application in dental practice. Proceedings of the 2009 ICME International Conference on Complex Medical Engineering.

[29] Antwi-Afari M.F., Li H., Yu Y., Kong L. (2018). Wearable insole pressure system for automated detection and classification of awkward working postures in construction workers. Autom. Constr..

[30] Zhang L., Diraneyya M.M., Ryu J., Haas C.T., Abdel-Rahman E. Automated monitoring of physical fatigue using jerk. Proceedings of the International Symposium on Automation and Robotics in Construction (ISARC).

[31] Donisi L., Cesarelli G., Coccia A., Panigazzi M., Capodaglio E.M., D’Addio G. (2021). Work-related risk assessment according to the revised NIOSH lifting equation: A preliminary study using a wearable inertial sensor and machine learning. Sensors.

[32] Aiello G., Certa A., Abusohyon I., Longo F., Padovano A. (2021). Machine Learning approach towards real time assessment of hand-arm vibration risk. IFAC-PapersOnLine.

[33] Zhao J., Obonyo E. (2021). Applying incremental Deep Neural Networks-based posture recognition model for ergonomics risk assessment in construction. Adv. Eng. Inform..

[34] Umer W., Li H., Yantao Y., Antwi-Afari M.F., Anwer S., Luo X. (2020). Physical exertion modeling for construction tasks using combined cardiorespiratory and thermoregulatory measures. Autom. Constr..

[35] Yu Y., Li H., Yang X., Umer W. Estimating construction workers’ physical workload by fusing computer vision and smart insole technologies. Proceedings of the International Symposium on Automation and Robotics in Construction (ISARC).

[36] Mudiyanselage S.E., Nguyen P.H.D., Rajabi M.S., Akhavian R. (2021). Automated Workers’ Ergonomic Risk Assessment in Manual Material Handling Using SEMG Wearable Sensors and Machine Learning. Electronics.

[37] Donisi L., Capodaglio E., Pagano G., Amitrano F., Cesarelli M., Panigazzi M., D’Addio G. Feasibility of Tree-based Machine Learning algorithms fed with surface electromyographic features to discriminate risk classes according to NIOSH. Proceedings of the 2022 IEEE International Symposium on Medical Measurements and Applications (MeMeA).

[38] Jacob D., Unnsteinsdóttir Kristensen I.S., Aubonnet R., Recenti M., Donisi L., Ricciardi C., Svansson H.Á.R., Agnarsdóttir S., Colacino A., Jónsdóttir M.K. (2022). Towards defining biomarkers to evaluate concussions using virtual reality and a moving platform (BioVRSea). Sci. Rep..

[39] Kristinsdóttir S., Þóra Þórisdóttir A., Björk Halldórsdóttir L., Magnúsdóttir G., Ingólfsdóttir B., Ingvarsson P.E., Helgason Þ. (2022). A novel reflex analysis of healthy and spinal cord-injured individuals. Current Directions in Biomedical Engineering.

[40] Lu M.L., Waters T.R., Krieg E., Werren D. (2014). Efficacy of the revised NIOSH lifting equation to predict risk of low-back pain associated with manual lifting: A one-year prospective study. Hum. Factors.

[41] Waters T.R., Baron S.L., Piacitelli L.A., Anderson V.P., Skov T., Haring-Sweeney M., Wall D.K., Fine L.J. (1999). Evaluation of the revised NIOSH lifting equation: A cross-sectional epidemiologic study. Spine.

[42] Spector J.T., Lieblich M., Bao S., McQuade K., Hughes M. (2014). Automation of workplace lifting hazard assessment for musculoskeletal injury prevention. Ann. Occup. Environ. Med..

[43] Press W.H., Teukolsky S.A. (1990). Savitzky-Golay smoothing filters. Comput. Phys..

[44] Murthy S.K. (1998). Automatic construction of decision trees from data: A multi-disciplinary survey. Data Min. Knowl. Discov..

[45] Sheng P., Chen L., Tian J. Learning-based road crack detection using gradient boost decision tree. Proceedings of the 2018 13th IEEE Conference on Industrial Electronics and Applications (ICIEA).

[46] Weinberger K.Q., Saul L.K. (2009). Distance metric learning for large margin nearest neighbor classification. J. Mach. Learn. Res..

[47] Al-Aidaroos K.M., Bakar A.A., Othman Z. Naive Bayes variants in classification learning. Proceedings of the 2010 International Conference on Information Retrieval & Knowledge Management (CAMP).

[48] Kotsiantis S.B., Zaharakis I., Pintelas P. (2007). Supervised machine learning: A review of classification techniques. Emerg. Artif. Intell. Appl. Comput. Eng..

[49] Stoltzfus J.C. (2011). Logistic regression: A brief primer. Acad. Emerg. Med..

[50] Anguita D., Ghelardoni L., Ghio A., Oneto L., Ridella S. The ‘K’ in K-fold Cross Validation. Proceedings of the European Symposium on Artificial Neural Networks, Computational Intelligence and Machine Learning (ESANN).

[51] Sechidis K., Tsoumakas G., Vlahavas I. (2011). On the stratification of multi-label data. Machine Learning and Knowledge Discovery in Databases, Proceedings of the European Conference, ECML PKDD 2011, Athens, Greece, 5–9 September 2011.

[52] Hossin M., Sulaiman M.N. (2015). A review on evaluation metrics for data classification evaluations. Int. J. Data Min. Knowl. Manag. Process.

[53] Lei S. A feature selection method based on information gain and genetic algorithm. Proceedings of the 2012 International Conference on Computer Science and Electronics Engineering.

[54] Petrofsky J., Laymon M. (2005). Muscle temperature and EMG amplitude and frequency during isometric exercise. Aviat. Space Environ. Med..

[55] Klein A.B., Snyder-Mackler L., Roy S.H., DeLuca C.J. (1991). Comparison of spinal mobility and isometric trunk extensor forces with electromyographic spectral analysis in identifying low back pain. Phys. Ther..

[56] Roy S.H., De Luca C.J., Casavant D.A. (1989). Lumbar muscle fatigue and chronic lower back pain. Spine.

[57] Kramer M., Ebert V., Kinzl L., Dehner C., Elbel M., Hartwig E. (2005). Surface electromyography of the paravertebral muscles in patients with chronic low back pain. Arch. Phys. Med. Rehabil..

[58] D’Addio G., Donisi L., Cesarelli G., Amitrano F., Coccia A., La Rovere M.T., Ricciardi C. (2021). Extracting features from Poincare plots to distinguish congestive heart failure patients according to NYHA classes. Bioengineering.

[59] Vispute S., Saini M.L. (2022). Performance Analysis of Soil Health Classifiers Using Data Analytics Tools and Techniques for Best Model and Tool Selection. Int. J. Online Biomed. Eng..

[60] Fillbrunn A., Dietz C., Pfeuffer J., Rahn R., Landrum G.A., Berthold M.R. (2017). KNIME for reproducible cross-domain analysis of life science data. J. Biotechnol..

[61] Balderston S., Taulbee J.J., Celaya E., Fung K., Jiao A., Smith K., Hajian R., Gasiunas G., Kutanovas S., Kim D. (2021). Discrimination of single-point mutations in unamplified genomic DNA via Cas9 immobilized on a graphene field-effect transistor. Nat. Biomed. Eng..

[62] Ricciardi C., Ponsiglione A.M., Scala A., Borrelli A., Misasi M., Romano G., Russo G., Triassi M., Improta G. (2022). Machine learning and regression analysis to model the length of hospital stay in patients with femur fracture. Bioengineering.

[63] Özkan S.B., Apaydin S.M.F., Özkan Y., Düzdar I. Comparison of open source data mining tools: Naive Bayes algorithm example. Proceedings of the 2019 Scientific Meeting on Electrical-Electronics & Biomedical Engineering and Computer Science (EBBT).

[64] Kamen G., Gabriel D.A. (2009). Essentials of Electromyography.

[65] Donisi L., Cesarelli G., Capodaglio E., Panigazzi M., D’Addio G., Cesarelli M., Amato F. (2022). A Logistic Regression Model for Biomechanical Risk Classification in Lifting Tasks. Diagnostics.

[66] Varrecchia T., De Marchis C., Rinaldi M., Draicchio F., Serrao M., Schmid M., Conforto S., Ranavolo A. (2018). Lifting activity assessment using surface electromyographic features and neural networks. Int. J. Ind. Ergon..

[67] Varrecchia T., De Marchis C., Draicchio F., Schmid M., Conforto S., Ranavolo A. (2020). Lifting Activity Assessment Using Kinematic Features and Neural Networks. Appl. Sci..

[68] Ranavolo A., Varrecchia T., Iavicoli S., Marchesi A., Rinaldi M., Serrao M., Conforto S., Cesarelli M., Draicchio F. (2018). Surface electromyography for risk assessment in work activities designed using the “revised NIOSH lifting equation”. Int. J. Ind. Ergon..

[69] Lindstrom L., Kadefors R., Petersen I. (1977). An electromyographic index for localized muscle fatigue. J. Appl. Physiol..

[70] Van Boxtel A., Goudswaard P., Van der Molen G.M., Van Den Bosch W.E. (1983). Changes in electromyogram power spectra of facial and jaw-elevator muscles during fatigue. J. Appl. Physiol..

[71] Bilodeau M., Schindler-Ivens S., Williams D.M., Chandran R., Sharma S.S. (2003). EMG frequency content changes with increasing force and during fatigue in the quadriceps femoris muscle of men and women. J. Electromyogr. Kinesiol..

[72] Löscher W.N., Cresswell A.G., Thorstensson A. (1994). Electromyographic responses of the human triceps surae and force tremor during sustained sub-maximal isometric plantar flexion. Acta Physiol. Scand..

[73] Modi S., Lin Y., Cheng L., Yang G., Liu L., Zhang W.J. (2011). A socially inspired framework for human state inference using expert opinion integration. IEEE/ASME Trans. Mechatron..

